# Estradiol-mediated inhibition of DNMT1 decreases p53 expression to induce M2-macrophage polarization in lung cancer progression

**DOI:** 10.1038/s41389-022-00397-4

**Published:** 2022-05-19

**Authors:** Yung-Ching Chen, Ming-Jer Young, Hui-Ping Chang, Chia-Yu Liu, Chia-Chi Lee, Yau-Lin Tseng, Yi-Ching Wang, Wen-Chang Chang, Jan-Jong Hung

**Affiliations:** 1 Institute of Basic Medical Sciences, National Cheng Kung University, Tainan, Taiwan; 2 Department of Biotechnology and Bioindustry Sciences, National Cheng Kung University, Tainan, Taiwan; 3 Division of Thoracic Surgery, Department of Surgery, College of Medicine National Cheng Kung University, Tainan, Taiwan; 4 Department of Pharmacology, College of Medicine, National Cheng Kung University, Tainan, Taiwan; 5 The Ph.D. Program for Neural Regenerative Medicine, College of Medical Science and Technology, Taipei Medical University, Taipei, Taiwan; 6 Graduate Institute of Medical Sciences, College of Medicine, Taipei Medical University, Taipei, Taiwan

**Keywords:** Non-small-cell lung cancer, Cancer genetics

## Abstract

Previous studies indicate that estrogen positively regulates lung cancer progression. Understanding the reasons will be beneficial for treating women with lung cancer in the future. In this study, we found that tumor formation was more significant in female EGFR^L858R^ mice than in male mice. P53 expression levels were downregulated in the estradiol (E2)-treated lung cancer cells, female mice with EGFR^L858R^-induced lung cancer mice, and premenopausal women with lung cancer. E2 increased DNA methyltransferase 1 (DNMT1) expression to enhance methylation in the TP53 promoter, which led to the downregulation of p53. Overexpression of GFP-p53 decreased DNMT1 expression in lung cancer cells. TP53 knockout in mice with EGFR^L858R^-induced lung cancer not only changed gene expression in cancer cells but also increased the polarization of M2 macrophages by increasing C–C motif chemokine ligand 5 (CCL5) expression and decreasing growth differentiation factor 15 (GDF15) expression. The TP53 mutation rate was increased in females with late-stage but not early-stage lung cancer compared to males with lung cancer. In conclusion, E2-induced DNMT1 and p53 expression were negatively regulated each other in females with lung cancer, which not only affected cancer cells but also modulated the tumor-associated microenvironment, ultimately leading to a poor prognosis.

## Introduction

Lung cancer, including non-small-cell lung cancer (NSCLC) and small-cell lung cancer (SCLC), is the leading cause of cancer-related death worldwide. Approximately 85% of all new cases are NSCLC, and adenocarcinoma is the most common subtype of NSCLC, especially in female patients. In the past three decades, the lung cancer incidence rate has decreased approximately twofold faster in men than in women. The clinical characteristics of men and women with lung cancer are very different. Previous studies have shown that when female patients were analyzed based on hormonal status, premenopausal women have a worse prognosis than both men and postmenopausal women, suggesting that estrogen promotes lung cancer malignancy in premenopausal women [1–3]. However, the detailed mechanism(s) remains to be elucidated.

TP53 is an important tumor suppressor gene that is involved in cell proliferation and metastasis through various mechanisms, such as the cell cycle and DNA damage repair [4, 5]. Previous studies have shown that p53 and RAS are involved in the regulation of cancer cell epithelial-mesenchymal transition (EMT) [6, 7]. p53 forms a complex with MDM2 and Slug to promote MDM2-mediated Slug degradation [8, 9]. In addition, previous studies have indicated that p53 positively regulates miR200 to silence ZEB1, subsequently increasing E-cadherin expression and decreasing Vimentin expression to inhibit EMT [10–12]. Recent studies have revealed that p53 is involved in regulating the inflammatory tumor microenvironment and maintaining cancer stem cells (CSCs) [13]. According to previous studies, p53 may be a regulator of the NF-κB signaling pathway [14, 15]. Several lines of evidence support the involvement of p53 in the inflammatory tumor microenvironment. First, the loss of p53 augments NF-κB transcriptional activity in response to TNF-α treatment. Second, the loss of p53 sensitizes cells to TNF-α-induced apoptosis. Third, the loss of p53 enhances inflammatory responses by inhibiting interleukin-1 receptor antagonist (sIL-1Ra) expression. Fourth, the loss of TP53 promotes TLR3-induced cytokine and chemokine expression. Finally, the loss of p53 affects the gene expression profile [16, 17]. In this study, we found that estrogen inhibited p53 expression and induced M2-macrophage polarization. The relationship between the loss of p53 and the tumor microenvironment in lung cancer needs to be elucidated.

DNA methylation is an important modification of the genome that modulate the gene expression and is involved in physiological and pathological conditions. Several enzymes, including DNMT1, DNMT3a, and DNMT3b, mediate DNA methylation [18–20]. A recent study indicates that inhibition of DNMT1 and ERα cross-talk suppresses breast cancer by inhibiting IRF4 expression [21]. In addition, DNMT1 increases the DNA methylation of TP53 and decreases p53 expression [22, 23]. DNMT1 promotes cell proliferation by methylating the hMLH1 and hMSH2 promoters in EGFR-mutated NSCLC. SUV39H1-mediated DNMT1 participates in the epigenetic regulation of Smad3 in cervical cancer [24, 25]. In this study, we found that estrogen-induced DNMT1 increased the DNA methylation of TP53 to repress p53 expression, which positively affected DNMT1 expression, thus promoting poor prognosis of lung cancer by modulating EMT and the microenvironment.

## Results

### E2-mediated inhibition of p53 is related to a poor prognosis in females with lung cancer

**Fig. 1:**
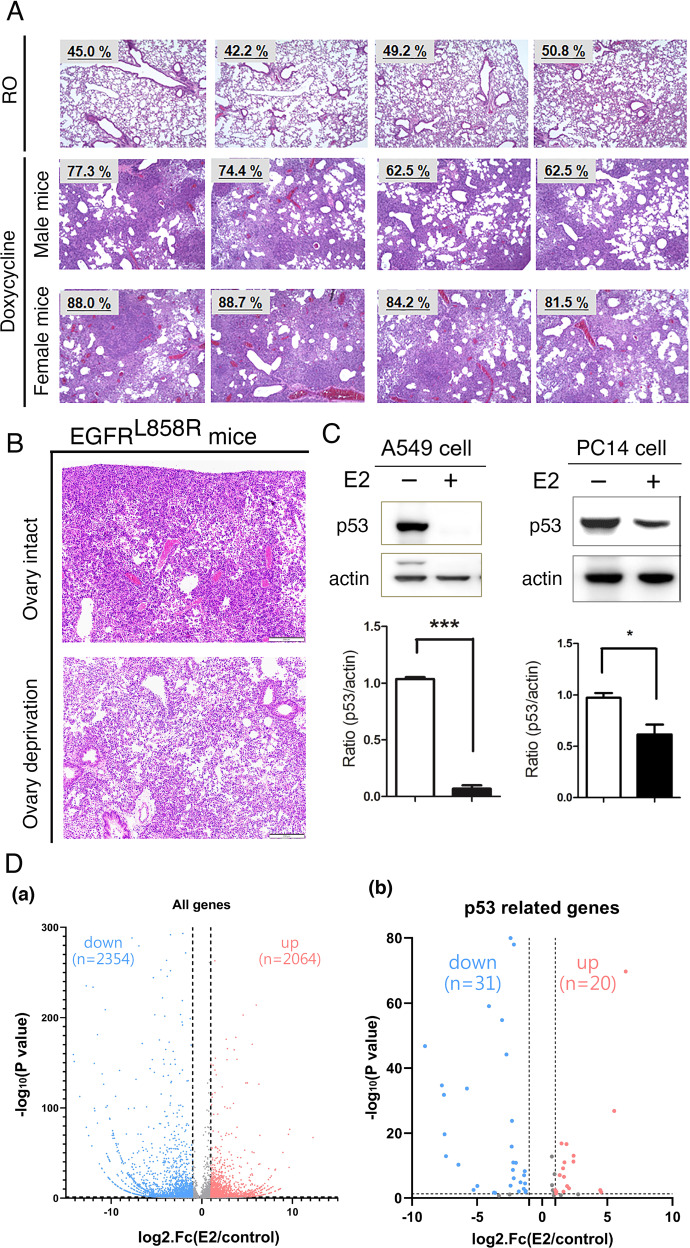
Estradiol (E2) decreases p53 expression and is related to lung cancer progression. Cancer development was induced by doxycycline treatment for 1.5 months in mice with EGFR^L858R^-induced lung cancer mice. The tumor areas in 12 mice including 4 control (RO, 2 male + 2 female), 4 male, and 4 female doxycycline-treated mice, were quantitated (**A**). The ovaries were removed from three female mice with EGFR^L858R^-induced lung cancer mice by ovariectomy, and mice were then sacrificed for H&E staining (**B**). A549 and PC14 cells with or without E2 treatment were used to study the level of p53 by IB with anti-p53 antibodies. After three independent experiments were completed, the level of p53 was quantified, and statistical assays were performed by a *t* tests; *P < 0.01, ***P < 0.001 (**C**). RNA samples were extracted from A549 cells with or without E2 treatment to study the global gene expression by RNA-sequencing assay. Up- and downregulated genes are listed (**D**, **a**); among these E2-regulated genes, p53-regulated genes are listed (**D**, **b**).

According to a previous study, estrogen may positively regulate lung cancer development [26–28], but the exact mechanisms need to be clarified. In this study, we used the tet-ON system, which is a well-known conditional transgenic system, to express EGFR^L858R^, which leads to cancer development [29, 30]. Herein, we first showed that lung cancer development was worse in female EGFR^L858R^-induced mice than in male mice (Fig. 1A) and ovariectomized female mice (Fig. 1B). However, the molecular mechanism by which E2 positively regulates lung cancer progression remains unclear. First, next-generation sequencing (NGS) of A549 and E2-A549 cells was performed to study the gene expression profiles (Fig. 1D). There were 2354 genes and 2064 genes that were negatively and positively regulated respectively, by E2 treatment in A549 lung cancer cells (Fig. 1D). The mRNA and protein levels of p53 were dramatically decreased in estrogen-treated A549 and PC14 cells (Fig. 1C,D). In addition, 51 p53-regulated genes, including 31 downregulated and 20 upregulated genes [31], were also regulated in E2-A549 cells, suggesting that E2-mediated inhibition of p53 regulated the expression of many p53 target genes during lung cancer progression (Fig. 1D and Supplementary Fig. 1). Taken together, these findings suggest that estrogen can decrease p53 expression by inhibiting its transcriptional activity in the early period of lung cancer progression.

**Fig. 2:**
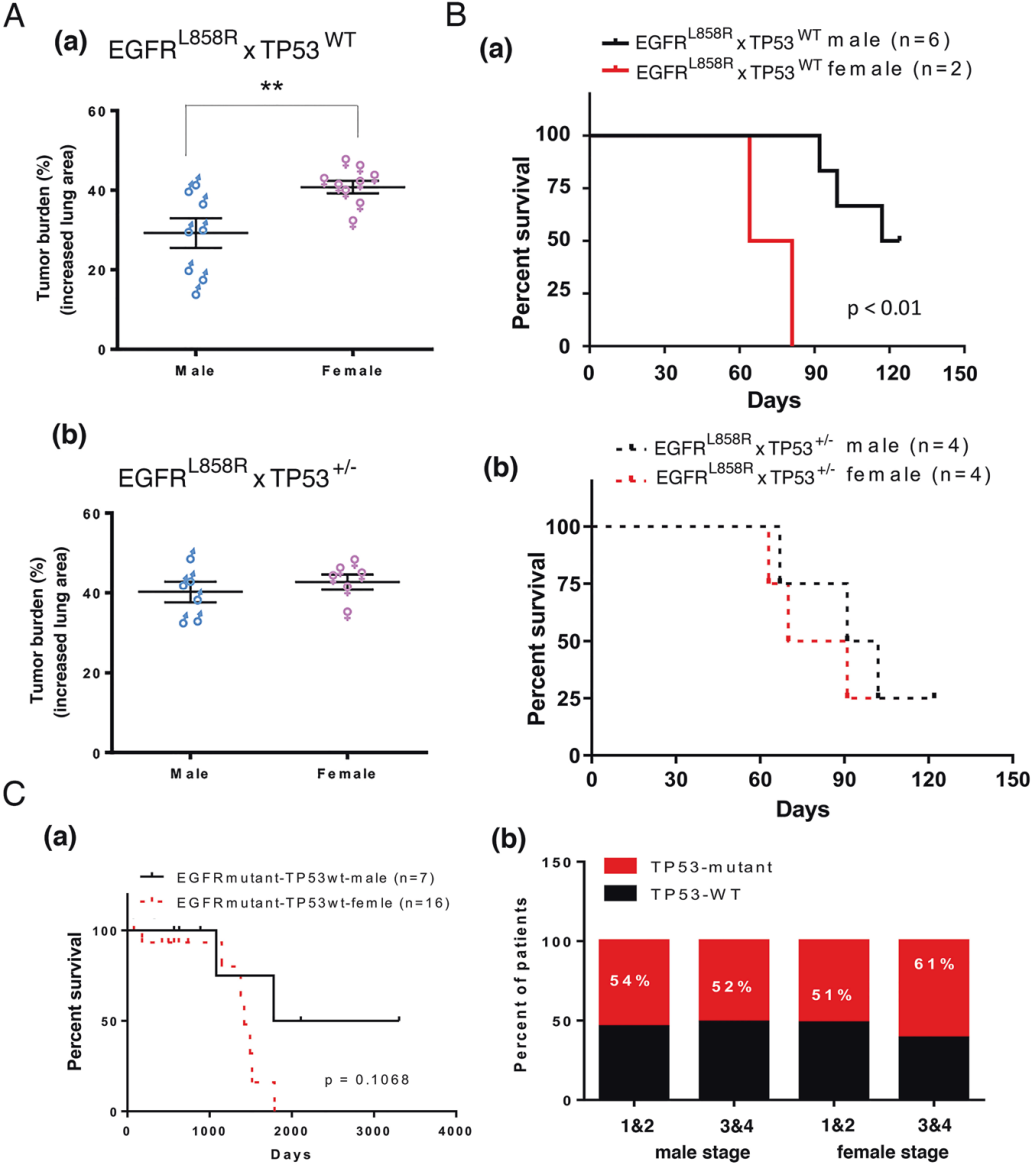
p53 is involved in sex-dependent lung cancer progression. The tumor areas in mice with EGFR^L858R^- and EGFR^L858R^ x TP53^−/+^-induced lung cancer were studied by H&E staining. All the H&E staining images are shown in Supplementary Fig. 2, and the lung areas were measured by ImageJ. A statistical assay was performed by a *t* tests; **P < 0.005. The tumor burden was defined as the increase in the lung area compared to the lung area in normal mice (**A**). Tumor formation in another 16 mice (8 EGFR^L858R^- and 8 EGFR^L858R^ × TP53^−/+^ mice) was induced by continuous doxycycline treatment until death. The survival rates of male and female EGFR^L858R^-induced mice with TP53 (**B**, **a**) or without TP53 (**B**, **b**) were examined by the Kaplan–Meier method. The TP53 mutation rates in male and female patients with lung cancer were collected from The Cancer Genome Atlas (TCGA). The survival rates of male and female lung cancer patients with EGFR mutations and wild-type TP53 were studied by the Kaplan–Meier method (**C**, **a**). The TP53 mutation rates in the early and late stages of lung cancer were analyzed (**C**, **b**).

To study the role of p53 in sex-dependent lung cancer progression, TP53 was knocked out in mice with EGFR^L858R^-induced lung cancer and EGFR^L858R^ x TP53^+/−^ male and female mice to delineate the role of p53 in sex-dependent lung cancer development induced by doxycycline for 1.5 months (Fig. 2A and Supplementary Fig. 2). The tumor burdens of doxycycline-induced lung cancer mice were determined by measuring the increase in the lung area compared to that of normal mice. The tumor burden in female mice was worse than that in male mice (Fig. 2A, a), but there was no significant difference in tumor size between male and female TP53-knockout mice, suggesting that the loss of p53 is the major reason for the poor prognosis in females with lung cancer mice (Fig. 2A, b and Supplementary Fig. 2). Cancer development in several mice was induced by continuous doxycycline until death to study the effect of TP53 mutations on survival rates. The survival rates of TP53-wt females were lower than that of TP53-wt male mice with EGFR^L858R^-induced lung cancer (Fig. 2B, a). However, there was no significant difference between TP53-knockout male and female mice with EGFR^L858R^-induced lung cancer (Fig. 2B, b). According to previous studies, the TP53 mutation rate is higher in women with lung cancer than in men [32, 33]. We examined the TP53 genotype in lung cancer cohorts from the TCGA database to analyze the TP53 mutation rate in the early and late stages of lung cancer (Fig. 2C and Supplementary Table 2). The survival rates of TP53-wt females were lower than that of TP53-wt males with EGFR mutations (Fig. 2C, a). Interestingly, in the early stage, there was no difference in the TP53 mutation rate between men and women with lung cancer. However, the TP53 mutation rate was higher in women with late-stage lung cancer than in men with late-stage men lung cancer (Fig. 2C, b). Based on these findings, we believe that estrogen decreases p53 expression in the early stage of lung cancer progression in women, subsequently inducing genomic instability and leading to TP53 mutation in the late stage, which ultimately results in a poor prognosis. In conclusion, these data indicate that E2-mediated inhibition of p53 in early-stage lung cancer may be related to the poor prognosis of women with lung cancer compared with men with lung cancer.

### E2-induced DNMT1 expression increases DNA methylation of p53 to inhibit its transcriptional activity

**Fig. 3:**
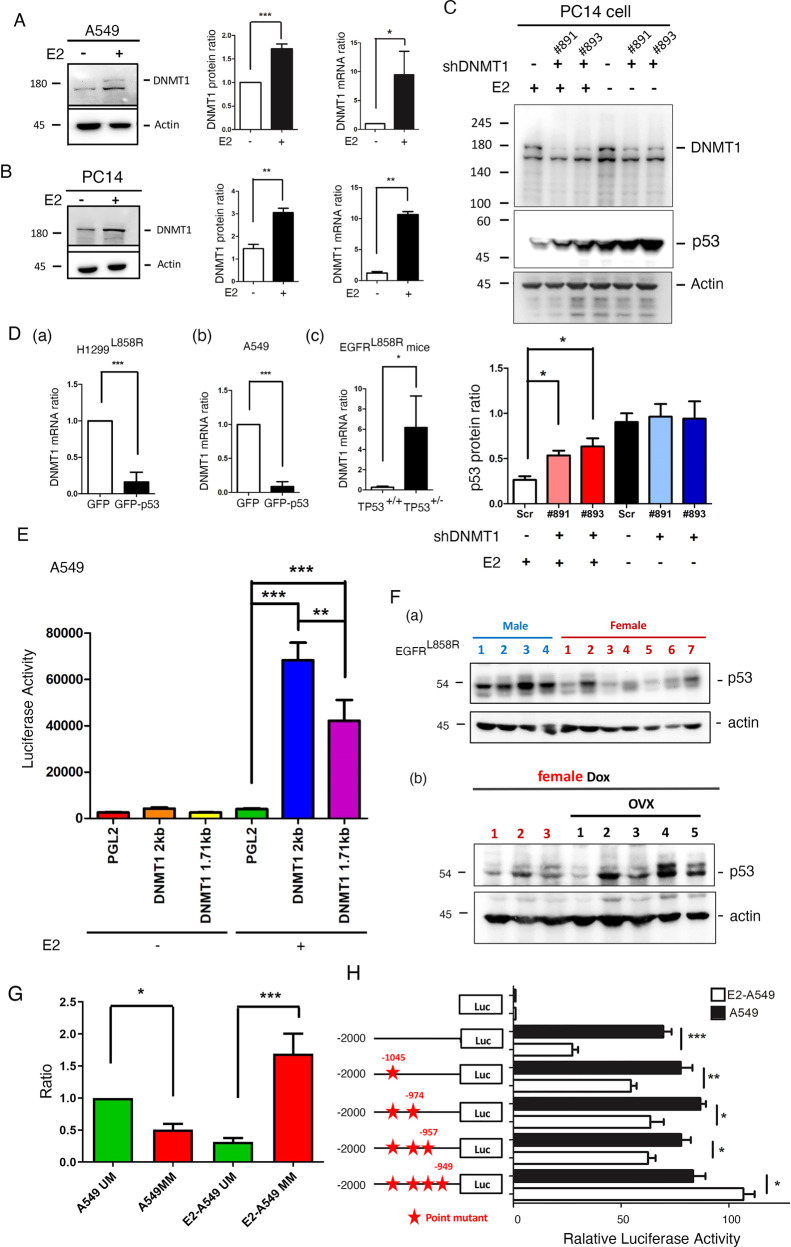
E2 induces DNMT1 expression to inhibit p53 expression by increasing DNA methylation in lung cancer. A549 (**A**) and PC14 (**B**) cells with or without E2 (1 mg/ml) treatment for 24 h were used to study the protein and mRNA levels of DNMT1 by IB with anti-DNMT1 antibodies and q-PCR, respectively. DNMT1 was knocked down by shRNA#1 and shRNA#2 in PC14 cells with or without E2 treatment for 24 h, and then the levels of DNMT1 and p53 were examined by IB with antibodies against the indicated proteins (**C**). Total RNA was extracted from H1299^L858R^ cells (**D**, **a**) and A549 cells (**D**, **b**) with or without GFP-p53 overexpression and from EGFR^L858R^-induced mice with or without TP53 knockout (**D**, **c**) to study the mRNA level of DNMT1 by q-PCR. Two promoter regions of DNMT1 (−1 to −2000 bp and −300 to −2000 bp) were cloned into the luciferase plasmid pGL2, and then 1 μg of pGL2, pGL2-DNMT1 promoter (2 Kbp) and pGL2-DNMT1 (1.7 Kbp) plasmids were transfected into A549 cancer cells with or without 1 μg/ml E2 treatment for 24 h to study luciferase activity (**E**). Samples from male and female mice with EGFR^L858R^-induced lung cancer (**F**, **a**), and female mice with or without ovariectomy (OVX) (**F**, **b**) were collected to study the level of p53 by IB with anti-p53 antibodies. Genomes were extracted from A549 cells with or without E2 treatment and used to study DNA methylation by q-PCR after 5-Fu treatment and then normalized to the level of GAPDH (**G**). The promoter of TP53 (−1 to −2000 bp) was cloned into a luciferase plasmid. Various methylation sites were individually mutated. The plasmids were transfected into A549 cells with or without E2 treatment to study the luciferase activity (**H**). After three independent experiments were completed, the results were quantified and statistical assays were performed by a *t* tests; *P < 0.05, **P < 0.01, ***P < 0.005.

A previous study indicates that DNA methylation in the TP53 promoter region is increased during cancer progression, thereby silencing p53 expression [23, 34]. Our NGS gene expression profile also showed that DNMT1 was increased in E2-treated A549 lung cancer cells (Supplementary Fig. 1). The mRNA and protein levels of DNMT1 were increased in E2-treated A549 and PC14 lung cancer cells (Fig. 3A, B). E2 treatment decreased the level of p53, and knockdown of DNMT1 increased the level of p53 in PC14 cells (Fig. 3C), suggesting that E2 increases DNMT1 to inhibit p53 expression. Because p53 can repress DNMT1 expression in colon cancer cells [35], here we also showed that the overexpression of GFP-p53 in H1299^L858R^ and A549 cells significantly decreased the level of DNMT1. Furthermore, knockout of TP53 in mice with EGFR^L858R^-induced lung cancer increased the level of DNMT1 (Fig. 3D, right panel). The promoter of DNMT1 was analyzed by software, and several estrogen receptor (ER)-binding sites were found in the promoter of DNMT1. To study the effect of E2 on the transcriptional activity of DNMT1, luciferase activity driven by the promoter of DNMT1 was studied (Fig. 3E). The data indicated that E2 treatment significantly increased luciferase activity in A549 cells (2.0-Kbp promoter) and the loss of one estrogen receptor (ER) binding site (1.7-Kbp promoter) decreased luciferase activity, indicating that estrogen directly binds with ER to induce the transcriptional activity of DNMT1 (Fig. 3E). In addition, the level of p53 in male lung cancer mice was higher than that in female lung cancer mice (Fig. 3F, a). To study the role of sex in p53 levels, the ovary was removed from female lung cancer mice by ovariectomy (Fig. 3F). The levels of p53 in male mice were higher than those in female mice. When the ovaries were removed, the levels of p53 in ovariectomized mice were greater than that in ovary-intact female lung cancer mice, indicating that estrogen is involved in the downregulation of p53 expression (Fig. 3F). Because DNMT1 is a DNA methyltransferase, the effect of estrogen treatment on the methylation of the TP53 promoter was studied (Fig. 3G). The data indicated that the methylation of the TP53 promoter region was increased by E2 treatment in A549 cells (Fig. 3G). To identify the detailed methylation site(s) within the TP53 promoter, the promoter activity of TP53 was studied by a luciferase activity assay (Fig. 3H). The data indicated that the luciferase activity of the TP53 promoter was significantly decreased by E2 treatment, but this effect was abolished by removing the methylation motifs of the TP53 promoter, especially mutating the −1045 and −949 sites, indicating that E2-induced DNMT1 enhancement of DNA methylation within the TP53 promoter is important for decreasing p53 expression (Fig. 3H). In summary, E2 treatment of lung cancer cells increases DNMT1 expression to enhance the DNA methylation of TP53, subsequently repressing p53 expression.

### E2-mediated inhibition of p53 increases the differentiation of M2 macrophages

**Fig. 4:**
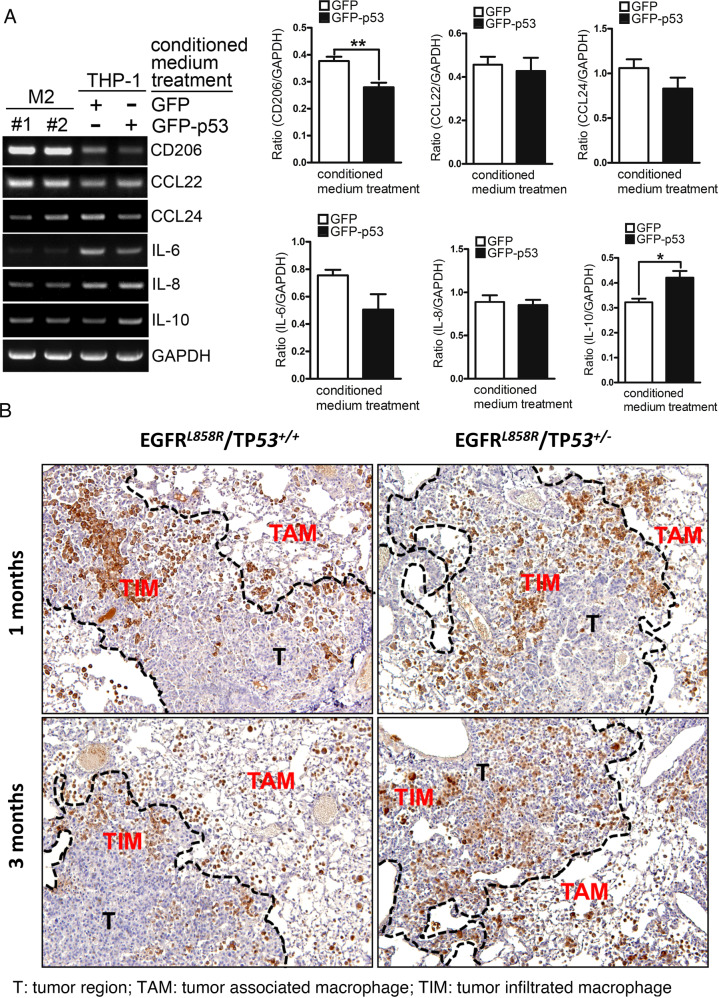
p53 regulates the tumor-associated microenvironment (TME) of lung cancer cells. Total RNA was extracted from M2 macrophages and THP-1 cells treated with conditioned medium collected from H1299^L858R^ cells with or without GFP-p53 overexpression to study the mRNA levels of the indicated genes by RT-PCR. After three independent experiments were completed, the mRNA levels were quantified and statistical assays were performed by a *t* tests; *P < 0.05, **P < 0.01 (**A**). The distribution of M2 macrophages in the tumor microenvironment of mice with EGFR^L858R^- and EGFR^L858R^ × TP53^−/+^-induced lung cancer that were treated with 10 mg/ml doxycycline in water for 1 month (**B**, upper panel) or 3 months (**B**, lower panel) was studied by immunohistochemistry (IHC) with anti-YM1 antibodies. T tumor region, TAM tumor-associated macrophage, TIM tumor-infiltrated macrophage (**B**).

**Fig. 5:**
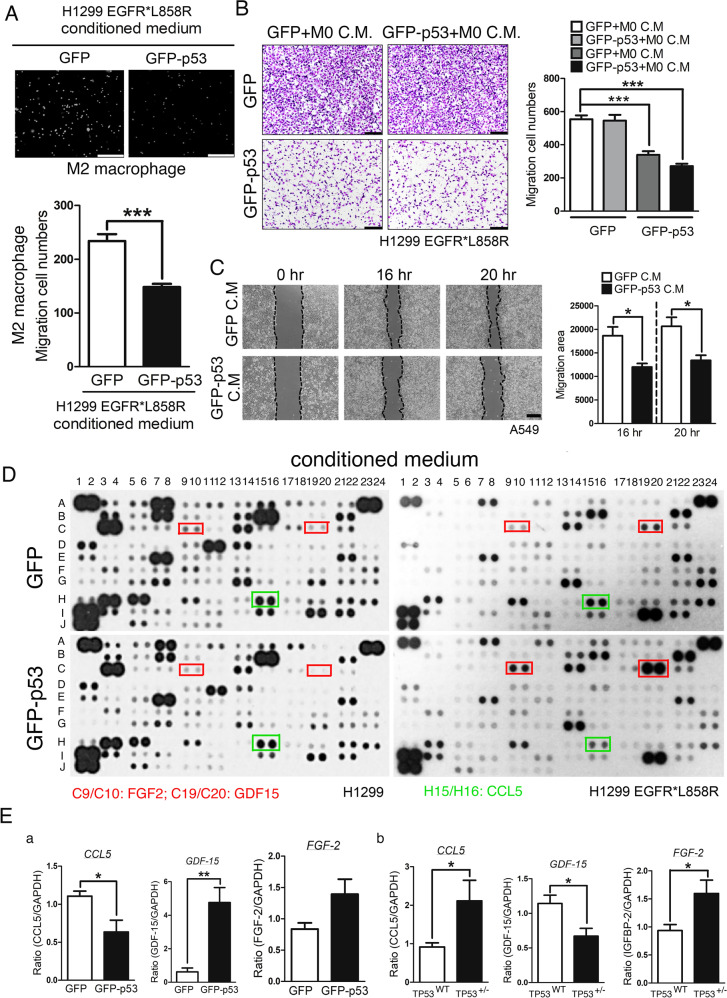
The p53-mediated changes in conditioned medium are involved in the migration abilities of macrophages and lung cancer cells. The M2 macrophages (**A**), H1299^L858R^ cells (**B**), and A549 cells (**C**) were treated with conditioned medium collected from H1299^L858R^ cancer cells with or without GFP-p53 overexpression; cell migration was studied by the chamber assay and wound healing assay. The conditioned medium collected from H1299 cells (**D**, left panel) and H1299^L858R^ cells (**D**, right panel) lung cancer cells with or without GFP-p53 overexpression were used to examine the protein repertoire by a protein array (**D**). The mRNA levels of CCL5, GDF15, and FGF2 in H1299^L858R^ cells with or without GFP-p53 overexpression (**E**, **a**) and in mice with EGFR^L858R^-induced lung cancer with or without TP53 knockout (**E**, **b**) were studied by q-PCR. After three independent experiments were completed, the statistical assay was performed by *t* tests; *P < 0.05, **P < 0.01, ***P < 0.005.

Because previous studies have indicated a higher TP53 mutation rate in women with lung cancer than in men with lung cancer, this might be the major reason for the poor prognosis in women. Many p53-regulated EMT- and cancer malignancy-related genes were changed by E2 treatment (Supplementary Fig. 1) [36], but the effect of p53 on the tumor-associated microenvironment (TME) in lung cancer needs to be addressed. To study the effect of p53 on TME during lung cancer progression, TP53 was knocked out in the EGFR^L858R^-, and EGFR^L858R^ × TP53^−/−^-induced lung cancer mouse models (Fig. 4). Tumor size was increased in TP53-knockout mice (Fig. 1 and Supplementary Fig. 2). Based on these results, we hypothesized that p53 regulates not only cancer cells but also other cells in the TME, such as macrophages. The conditioned medium of H1299^L858R^ cells with or without GFP-p53 expression was collected and then cultured with THP-1 monocytes to study the polarization of macrophage M1/M2 macrophages (Fig. 4). The data indicated that THP-1 cells treated with conditioned medium from H1299^L858R^ lung cancer cells with GFP-p53 overexpression decreased the levels of CD206 and IL6 but increased the level of IL10, suggesting that p53 in lung cancer cells can inhibit the polarization of M2 macrophages, which can promote cancer metastasis (Fig. 4A). We used the M2-macrophage marker YM1 to study the distribution of M2 macrophages around lung tumors in EGFR^L858R^ and EGFR^L858R^ × TP53^+/−^ mice (Fig. 4B). Interestingly, the tumor infiltration of M2 macrophages (TIMs) was markedly increased in TP53-knockout mice, suggesting that the loss of p53 facilitated macrophage infiltration in the lung cancer region, which might be beneficial for cancer progression (Fig. 4B). The effect of p53-mediated secreted factors on the migration of macrophages and cancer cells was studied (Fig. 5A–C). Conditioned medium from H1299^L858R^ cells overexpressing GFP-p53 inhibited the migration of macrophages (Fig. 5A) and cancer cells, H1299^L858R^ (Fig. 5B) and A549 cells (Fig. 5C), suggesting that p53-mediated secreted factors from lung cancer cells are involved in lung cancer progression by regulating cancer cells and the tumor-associated microenvironment. To study the composition of the conditioned medium, protein arrays were used to examine the conditioned medium of H1299 and H1299^L858R^ cells with or without GFP-p53 expression (Fig. 5D). Because we used mice with EGFR^L858R^-induced lung cancer to study the effect of p53 on the TME, in the protein array, we examined the differences in H1299^L858R^ but not in H1299 lung cancer cells (Fig. 5D). Only the proteins FGF2, GDF15, and CCL5 were regulated by p53 in H1299^L858R^ cells but not in H1299 cells. GFP-p53 overexpression increased the levels of FGF2 and GDF15 but decreased CCL5 in H1299^L858R^ cells (Fig. 5D). In addition, several proteins, including VEGF, MMP9, and PTX3, were decreased in GFP-p53-expressing H1299 and H1299^L858R^ cells, suggesting that these proteins are regulated by p53 in an EGFR mutation-independent manner (Fig. 5D and Supplementary Fig. 3). Furthermore, we validated the expression of CCL5, GDF15, and FGF2 in GFP-p53-expressing H1299^L858R^ cells (Fig. 5E, a) and EGFR^L858R^ x TP53^+/−^ mice (Fig. 5E, b). In H1299^L858R^ cells, GFP-p53 inhibited the mRNA level of CCL5 but increased the mRNA levels of FGF2 and GDF15, which was consistent with the conditional profile shown in Fig. 5D. In EGFR^L858R^ × TP53^+/−^ mice, the loss of p53 increased the mRNA level of CCL5 and decreased the mRNA level of GDF15, which was also consistent with the conditional profile. However, the loss of p53 increased the mRNA level of FGF2 (Fig. 5E, b, right panel), which was inconsistent with the results shown in Fig. 5D. Taken together, these results suggest that p53-mediated CCL5 and GDF15 are secreted by cancer cells to regulate the TME.

### p53-mediated CCL5 and GDF15 regulate M2-macrophage polarization to control cancer progression

**Fig. 6:**
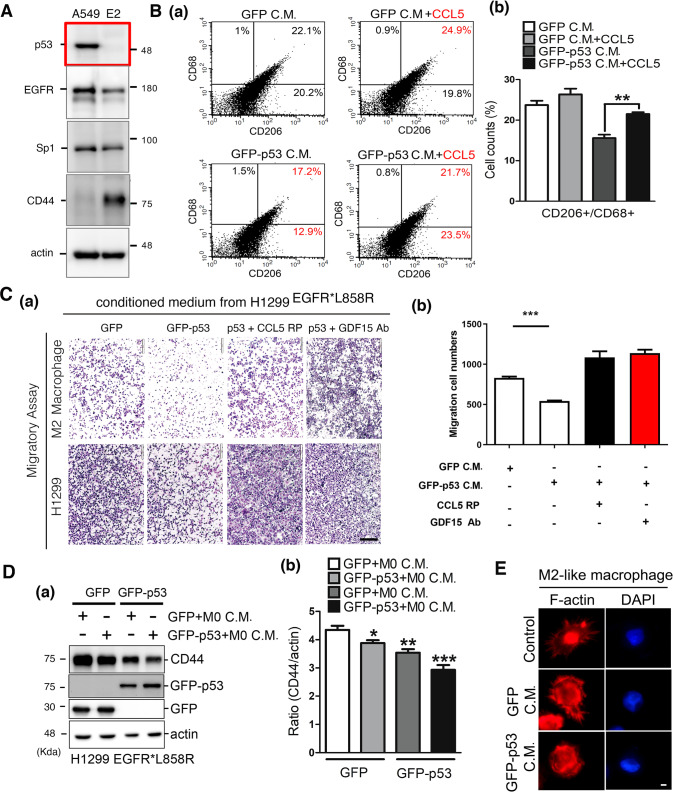
E2-mediated inhibition of p53 regulates CCL5 and GDF15 to modulate macrophage differentiation. The levels of p53, EGFR, Sp1, CD44, and actin in A549 and E2-treated A549 cells were studied by western blotting with the indicated antibodies (**A**). THP-1 cells were treated with conditioned medium from H1299^L858R^ cells with or without GFP-p53 expression and recombinant CCL5 protein treatment; subsequently, the differentiation of M2 macrophages was studied by flow cytometry with anti-CD68 and anti-CD206 antibodies (**B**). The migratory abilities of M2 macrophages (**C**, **a**, upper panel) and H1299 lung cancer cells (**C**, **a**, lower panel) in the presence or absence of conditioned medium from H1299^L858R^ cells with or without GFP-p53 expression and treated with recombinant CCL5 protein (RP) or a GDF15 antibodies were studied by chamber assays (**C**). The levels of CD44, GFP-p53, GFP, and actin in H1299^L858R^ cells with or without GFP-p53 expression were studied by Western blotting (**D**). The morphology of M2 macrophages in the presence or absence of conditioned medium from H1299^L858R^ cells with or without GFP-p53 expression was studied by immunofluorescent assays with anti-F-actin antibodies (**E**). After three independent experiments were completed, the results were quantitated, and the statistical assays were performed by *t* tests; *P < 0.05, **P < 0.01, ***P < 0.005.

**Fig. 7:**
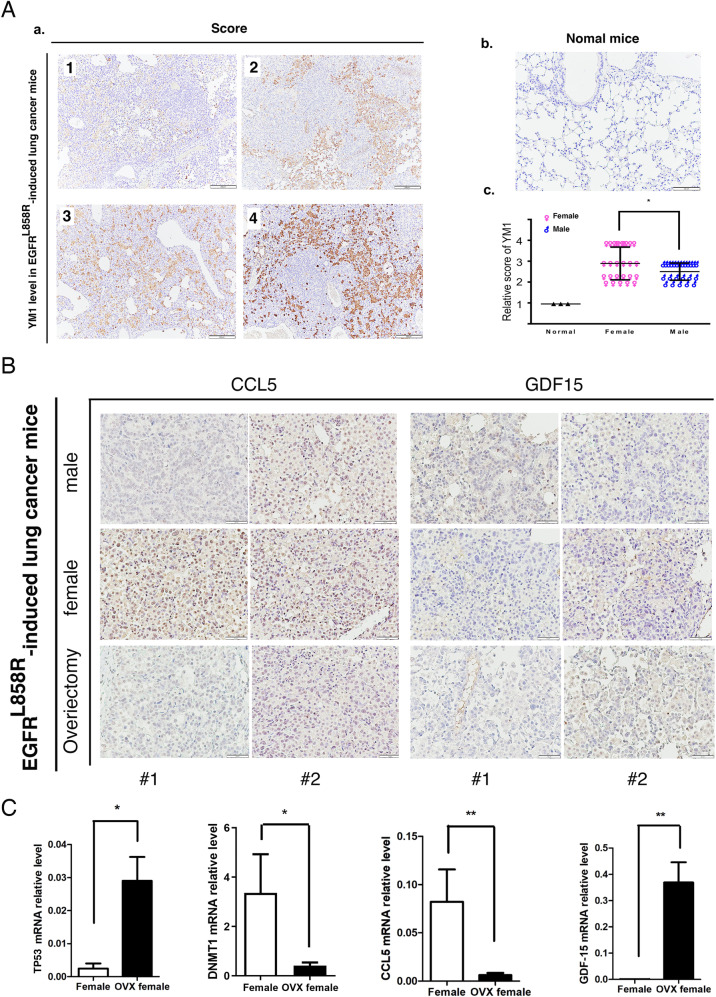
The relationship among the protein levels of YM1, CCL5, and GDF15 and sex-dependent changes in lung cancer in EGFR^L858R^ mice. The levels of YM1 were determined in EGFR^L858R^ mice, and scored based on the YM1 image (score 1 to 4) (**A**, **a**) compared to normal mice (**A**, **b**). All the scores were calculated in normal mice and mice with EGFR^L858R^-induced lung cancer, including female and male mice (**A**, **c**). The levels of CCL5 and GDF15 in mice with EGFR^L858R^-induced lung cancer mice, including male and female mice with or without ovariectomy, were studied by IHC with antibodies against the indicated proteins (**B**). Total RNA was extracted from mice with EGFR^L858R^-induced lung cancer with or without ovariectomy, and then the mRNA levels of p53, DNMT1, CCL5, and GDF15 were studied by q-PCR (**C**). After three independent experiments were completed, the quantitation and statistical analysis were performed with a *t* tests; *P < 0.05, **P < 0.01.

**Fig. 8:**
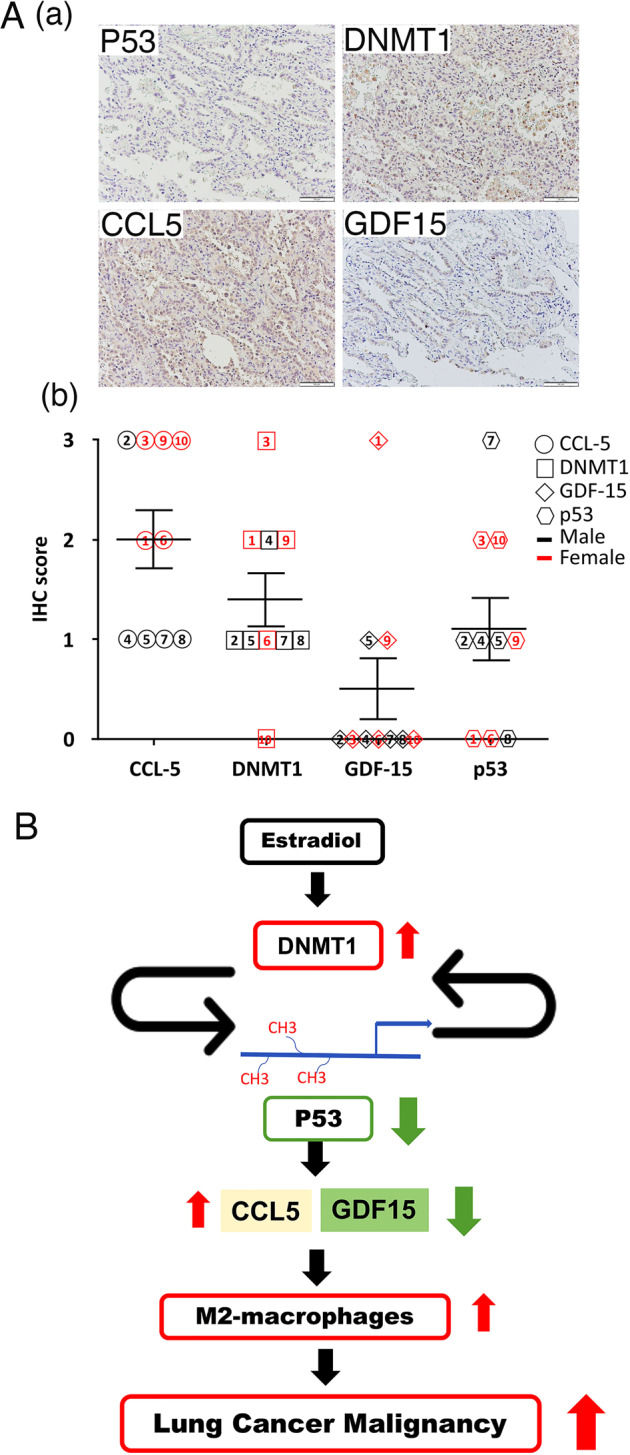
Clinical relevance of the levels of p53, DNMT1, GDF15, and CCL5 in lung cancer cohorts. The ten clinical specimens were collected from early-stage lung cancer patients used to study the association among the levels of p53, DNMT1, GDF15, and CCL5 by IHC staining (**A**, **a**), and the results were scored from 0 to 3 according to the IHC signal (**A**, **b**). The working model of the role of estradiol in lung cancer malignancy is shown (**B**).

What is the effect of the p53-mediated TAM on cancer progression? First, we found that E2 treatment of A549 cells dramatically decreased p53 expression and increased CD44 expression in lung cancer cells and M2 macrophages, indicating that E2-mediated inhibition of p53 expression may lead to cancer malignancy (Fig. 6A). We used CD206 and CD68 as the M2-macrophage markers to study the effect of p53-induced CCL5 on M2-macrophage polarization (Fig. 6B). Conditioned medium collected from H1299^L858R^ cells with GFP-p53 overexpression decreased M2-macrophage polarization, but adding recombinant CCL5 protein to the conditioned medium reversed this effect (Fig. 6B), indicating that p53-mediated inhibition of CCL5 could prevent the differentiation of M2 macrophages. In addition, the effect of CCL5 and GDF15 on the migration of M2-macrophage and H1299 cancer cells was studied by culturing the cells with the conditioned medium collected from H1299^L858R^ cells with or without GFP-p53 overexpression (Fig. 6C). The data indicated that GFP-p53 overexpression inhibited the migration of M2 macrophages and H1299 lung cancer cells, and the addition of anti-GDF15 antibodies or recombinant CCL5 protein blocked the effect of GFP-p53 expression on cancer cell migration (Fig. 6C). When we used conditioned medium from H1299^L858R^ cells with p53 expression to treat H1299^L858R^ cancer cells, CD44 expression in cancer cells was inhibited, suggesting that 53 mediated the TAM to inhibit cancer stemness (Fig. 6D). The effect of p53-mediated changes in conditioned medium on the morphology of macrophages was also observed (Fig. 6E). The data indicated that lamellipodia around M2 macrophages were observed in control and GFP-overexpressing cells but were lost in GFP-p53-overexpressing cells, suggesting that p53 in lung cancer cells regulates secreted proteins to affect the morphology of macrophages, which is related to tumor infiltration (Fig. 6E). Finally, to study the association between M2 macrophages and sex-dependent lung cancer, samples were collected from male and female mice with EGFR^L858R^-induced lung cancer and used to study M2 macrophages that were stained with anti-YM1 antibodies and then scored from 1 to 4 based on the YM1 signal (Fig. 7A, a, b). The data indicated that the scores of female mice were higher than those of male mice, indicating that more M2 macrophages were found in female lung cancer mice (Fig. 7A). The level of CCL5 was increased in female lung cancer mice but was decreased after the ovary was removed (Fig. 7B, left panel). In addition, the level of GDF15 was slightly decreased in female lung cancer mice but was slightly increased after the ovary was removed (Fig. 7B, right panel). In addition, the mRNA levels of TP53 and GDF15 were increased, and the mRNA levels of DNMT1 and CCL5 were decreased in ovariectomized female mice, which is consistent with the results of this study (Fig. 7C). Finally, the levels of p53, DNMT1, GDF15, and CCL5 in lung cancer patients were studied (Fig. 8 and Supplementary Table 3). Samples from ten lung cancer patients were used to study the levels of p53, DNMT1, CCL5, and GDF15 and then scored from 0 to 3 based on protein signals (Fig. 8A). The data indicated that the scores of CCL5 and DNMT1 were higher than those of p53 and GDF15 (Fig. 8A) in the clinical samples from lung cancer patients, which is consistent with our study. In addition, the levels of CCL5 and DNMT1 in female patients were higher than those in male patients, but there was no significant difference in GDF15 and p53 levels between the male and female cohorts (Fig. 8A).

## Discussion

The poor prognosis of women with lung cancer has recently become an important clinical issue. In this study, we found that E2 inhibited p53 to control not only in cancer cells but also cells in the tumor-associated microenvironment. We found that E2-induced DNMT1 and p53 formed a negative feedback loop, thereby increasing CCL5 and decreasing GDF15 expression, subsequently enhancing M2-macrophage polarization and ultimately leading to poor prognosis in females with lung cancer (Fig. 8B).

Previous studies have revealed that E2 treatment induces rapid activation of the EGFR pathway, suggesting that nonnuclear ER translocation regulates the EGFR pathway to influence lung cancer progression [37]. Moreover, 67% of EGFR mutation-positive tumors have high nuclear ERβ expression (versus 37% in EGFR wild-type tumors) [38]. Based on these previous studies, ER-mediated and EGFR-mediated signaling pathways may positively coregulate the cancer-related gene expression during lung cancer progression. In this study, we found that E2 treatment could induce DNMT1 expression, subsequently increasing DNA methylation in the promoter of p53. Because E2 actives EGFR signaling and the TP53 promoter sequence contains ER-binding motifs, ERs may regulate the transcriptional activity of DNMT1 by activation of the EGFR signaling pathway and direct recruitment to its promoter.

According to previous studies, the TP53 mutation rate is higher in women with lung cancer than in men and is related to a poor prognosis in these individuals [32, 33]. In this study, we found high TP53 mutation rates only in females with late-stage lung cancer, not in those with early-stage lung cancer. We also found that estrogen significantly inhibited p53 expression and was an important factor in inducing the high TP53 mutation rate in late-stage lung cancer. p53, which is a tumor suppressor, maintains genomic integrity to avoid cancer heterogeneity during cancer progression [39, 40]. In this study, we found that the survival rates of EGFR^L858R^ male mice were higher than those of female mice, but this difference was abolished in EGFR^L858R^ × TP53^+/−^ mice, indicating that estrogen-mediated inhibition of p53 in female mice is critical for poor prognosis. Although we have studied the role and mechanism by which p53 affects tumor burden in vivo, we only used eight EGFR^L858R^ and EGFR^L858R^ × TP53^+/−^ mice to study the survival rate. More mice will be used to confirm this finding in the future. We also found that many p53 target genes were altered by estrogen treatment, which is consistent with previous studies in which p53 was revealed to be a tumor suppressor. In addition to the effect of p53 within cancer cells, when the TP53 was knocked out in mice with EGFR^L858R^-induced lung cancer, not only did the size of the tumor increase, but marked immune cell infiltration was also observed around cancer cells, indicating that p53, as a tumor suppressor, inhibited not only cancer cells directly but also M2-macrophage differentiation. The TAM is involved in cancer progression (i.e., cancer malignancy and drug resistance) [41–44]. Many proteins, such as cytokines secreted from nearby cells (e.g., macrophages, fibroblasts, and other immune cells, including T cells and B cells), regulate cancer cell progression and macrophage polarization. Our data indicated that estrogen-mediated inhibition of p53 in H1299^L858R^ cancer cells changed the protein composition (FGF2, GDF15, IGFBP2, CCL5, PTX3, VEGF, and MMP9) of conditioned medium. GDF15, CCL5, and FGF2 were regulated only in H1299^L858R^ cells but not in H1299 cancer cells, suggesting that EGFR signaling and p53 coregulate these three secreted proteins. Fibroblast growth factor type 2 (FGF2) can bind to heparin and has broad mitogenic and angiogenic activities. Our protein array data showed that GFP-p53 positively regulated FGF2 secretion by cells, and positively regulated FGF2 in cancer cells. However, FGF2 expression was also increased in TP53-knockout mice with EGFR^L858R^-induced lung cancer, which is inconsistent with the results in cancer cells. p53 may regulate FGF2 differently under different conditions, and the detailed regulatory mechanism needs to be further clarified. Growth differentiation factor 15 (GDF15) has been reported to be involved in various physiologic or pathologic pathways including cancer [45]. GDF15 is one of the ligands that bind to TGF-β receptors to modulate the SMAD pathway and regulate gene expression [46]. Previous studies have indicated that EZH2-mediated inhibition of GDF15 represses NSCLC proliferation [47]. In this study, we first showed that p53 positively regulated GDF15 to repress the migration abilities of M2 macrophages and lung cancer cells. In addition, C–C motif chemokine ligand 5 (CCL5) is a secreted protein involved in immunoregulatory and inflammatory processes that functions as a chemoattractant for blood monocytes, memory T-helper cells, and eosinophils [48–50]. Previous studies have indicated that IL6 mediates macrophage infiltration after irradiation by upregulating CCL5 expression in NSCLC. Our study showed that GFP-p53 increased GDF15 expression but decreased CCL5 expression in H1299^L858R^ cells, thereby repressing M2-macrophage differentiation and subsequently preventing cancer malignancy. E2-mediated EGFR signaling and p53 expression are critical for the poor prognosis in females with lung cancer.

DNA methylation is important for maintaining genomic integrity and gene expression [51, 52]. Disorders associated with DNA methylation are involved in various diseases, including cancer [53]. A previous study indicated that DNMT1 could methylate the promoter of TP53 to inhibit its expression [34, 54]. We showed that estrogen treatment of lung cancer cells could induce DNMT1 expression, thereby inducing hypermethylation of the TP53 promoter and leading TP53 downregulation. Our study also showed that other DNMTs, DNMT2, DNMT3a, and DNMT3b were slightly increased, which might also contribute to the hypermethylation of the TP53 promoter. Although there was no significant difference in TP53 mutations between male and female lung cancer patients in the early stage, estrogen-induced DNMT expression to inhibit p53 expression, which might be one of the critical reasons for the induction of genomic instability, subsequently increasing TP53 mutation rates in the late stage of lung cancer in female patients. In the future, blocking estrogen or DNMT activities may be beneficial for the survival rates of female lung cancer patients.

## Materials and methods

### Cell culture and transfection

Human lung adenocarcinoma epithelial cell line A549, PC14, H1299, H1299^L858R^, and human monocyte THP-1 cell lines from ATCC were cultured with RPMI 1640 medium (Invitrogen, Carlsbad, CA, USA) containing 10% fetal bovine serum (Gibco™, Waltham, MA, USA), 100 μg/ml streptomycin and 100 U/ml penicillin G sodium (Gibco). E2-A549 cells were obtained by treating A549 cells with 1 μg/ml E2 for 3 weeks. All cells were incubated at 37 °C with 5% CO_2_. For transfecting plasmid, PolyJet (SignaGen Laboratories, Frederick, MD, USA) was used according to the manufacturer’s instructions.

### Immunohistochemistry

Paraffin-embedded human and mice lung cancer tissues were obtained from the National Cheng Kung University Hospital Tissue Bank and doxycycline-induced EGFR^L858R^ mice, respectively, and the tissues were cut into 5-μm sections. Immunohistochemistry was performed using a Novolink™ Polymer Detection Systems (Leica Biosystems) following the manufacturer’s instructions. Antigen retrieval was performed using citrate buffer (pH 6.0, Scytek). Primary antibodies, anti-p53 (A5761, ABclonal, 1:50), anti-CCL5 (A5630, ABclonal, 1:50), anti-YM1 (Abcam, 1:50), anti-DNMT1 (A16729, ABclonal, 1:50) and anti-GDF15 (A0185, ABclonal, 1:50), were used to incubate with tissue samples for overnight at 4 °C. Sections were photographed by Olympus BX-51 microscope.

### Animal care and animal models

The experiments related with animals were approved by the Institutional Animal Care and Use Committee (IACUC:108005) at National Cheng Kung University (NCKU). These transgenic mice were generated in National Laboratory Animal Center (NLAC, Taiwan, Tainan). After breeding, two-month-old transgenic mice were used to study lung cancer development. Caging was provided suitable space and accommodates appropriate population densities that allowed animals’ sufficient freedom of movement. To provide amounts of food that must be for transgenic mice normal growth and maintenance of normal body weight. These transgenic mice, including EGFR^L858R^ and EGFR^L858R^ × TP53^+/−^ mice, were accessed to fresh and uncontaminated drinking water. Transgenic mice were also observed and cared at least for two to three times per week. All methods involving animals were performed in accordance with the relevant guidelines and regulations.

### Collection of specimens from lung cancer patients

All human study has been conducted in accordance with the guidelines and regulations. The study using human specimens was approved by the Clinical Research Ethics Committee at National Cheng Kung University Medical Center (Tainan, Taiwan; IRB: A-ER-107-039). After surgical resection at National Cheng Kung University Hospital, specimens of patients with lung adenocarcinomas were collected for Immunohistochemical analysis or western blotting. The pathological data were analyzed by clinical pathologists. Informed consent was obtained from all subjects.

### Statistical analysis

All samples were used for statistical analysis. The difference between two groups was analyzed by Student’s *t* test. The *P* value, which is smaller than 0.05, was considered statistically significant. SEM is used to calculate and plot error bars from raw data. Overall survivals were estimated by means of the Kaplan–Meier method and compared using the log-rank test.

## Supplementary information

Supplementary Materials
