# Supplementary material for: Estradiol-mediated inhibition of DNMT1 decreases p53 expression to induce M2-macrophage polarization in lung cancer progression
Source: Oncogenesis. 2022 May 19;11(1):25. doi: 10.1038/s41389-022-00397-4 (PMC9119954; doi:10.1038/s41389-022-00397-4)
Supplement: Supplementary file 1 — Supplementary Materials [file 41389_2022_397_MOESM1_ESM.docx]

**Supplementary information**

**Supplementary Materials and Methods**

**Lentivirus knockdown system** - Scramble knockdown and DNMT1 knockdown lentivirus were generated from RNAi core facility of Academia Sinica (Taiwan). Cells were seeded in 6-well-plates and incubated for 16 hours, and then treated with 1 ml RPMI medium containing 10 μg Polybrene (Millipore) and lentivirus with 5 Multiplicity of infection (MOI). After 24 hours of infection, medium containing lentivirus was replaced with fresh medium and maintained for another 72 hours.

**Western blotting**

Cells were collected by sample buffer and analyzed by electrophoresis. Proteins were transferred to polyvinylidene difluoride (PVDF, Millipore) membrane and TBST buffer (10 mM Tris-HCl, pH 8.0, 150 mM NaCl and 0.05% Tween 20) containing 5% nonfat milk was used for blocking anti-actin (Cat# 110564, Genetex, 1:20000), anti-GFP (Cat# sc-9996, Santa Cruz, 1:5000), anti-p53 (Cat# 05-224, Millipore, 1:3000), anti-ERβ, anti-CCL5, anti-GDF15, anti-CD44, anti-β-catenin, anti-ALDH1, anti-vimentin, anti-E-cadherin and anti-Sox2 were used for probing interested proteins. After incubated with primary antibodies, PVDF membranes were then incubated with secondary immunoglobulin antibodies linked with horse radish peroxidase (Millipore, 1:10,000). ECL Western blotting detection system (Millipore) and ChemiDoc-it imager (UVP) were used for detecting signals.

**Luciferase reporter assay**

8x10^4^ A549 cells and E2-treated A549 (E2-A549) cells were seeded in each well of 6-well plates for 16 hours. Reporter plasmids containing TP53 promoter region were transfected into A549 cells. Reporter assays were performed by using Dual-luciferase reporter assay system (Promega, Madison, USA) following manufacturer’s instruction. All the primers used in this study for plasmid construction and RT-PCR were listed in Supplementary Table 1.

**Wound healing and Chamber assay**

H1299 cells were maintained in 6 cm dishes till 60% density and after overexpression of GFP, GFP-p53 in cells for 24 hours, and then cells were scratched with 200-μl-tips. Cells were washed with PBS and photographed under microscopy observation. After incubation for another 24 hours at 37 °C, migrated distance then was measured and relative migrated distance was analyzed [1]. The cell migration assay was performed using Transwell Chamber (Corning^®^ Costar^®^, Sigma-Aldrich, St. Louis, MO, USA) with an 8-μM pore size poly carbonate filter membrane. After overexpression of GFP, GFP-p53 in H1299 and A549 cells for 24 hours, cells were trypsinized and suspended in serum-free RPMI 1640 medium. Lower wells were filled with RPMI 1640 medium containing 10% FBS and upper wells were filled with cell suspensions (2 × 10^4^) in serum-free RPMI 1460 medium. After incubation at 37 °C for 6 hours, the filter membrane on the lower side was fixed with 10% methanol and stained with DAPI for 3 minutes. Images of migrated cells were photographed by fluorescence Olympus BX-51 microscopy and migrated cell number were analyzed by ImageJ [1].

**Fluorescence-activated cell sorting (FACS)**

THP1 cells were adherent with PMA (100 ng/ml) (Merck KgAa, Germany). IL13/IL4 (PeproTech, Rocky Hill, NJ, USA) or condition medium (GFP/GFP-p53) treatment for 24 hours. Removed IL13/IL4 or condition medium (GFP/GFP-p53) and then cultured for 4 days. On day 4, cells were washed with PBS and fixed with 70% alcohol in 4°C for 16 hours. Cells were then incubated in cold PBS with 0.1% Triton X-100 for 10 minutes for permeabilization. Permeabilized cells were treated with 10 μg/ml RNase A (Qiagen, Germantown, MD, USA), and 100 μg/mL of CD206 (BioLegend, San Diego, CA, USA) and CD68 (BioLegend) in PBS at room temperature for 1 hour. Stained cells then analyzed by Cell Lab Quanta SC flow cytometry (Beckman Coulter, Brea, CA, USA).

**Ovariectomy surgeries**

Mice with 8 weeks of age underwent either ovariectomy or sham surgery under anesthesia induced by intraperitoneal injection of ketamine (100 mg/kg) and xylazine (7 mg/kg). Ovariectomy surgeries involved bilateral flank incisions through the skin and muscle wall and the removal of ovaries. Sham surgeries involved bilateral flank incisions through the skin and muscle wall. Incisions were closed using sterile 4-0 ETHILON nylon sutures. Buprenorphine (0.375 mg/kg) was administered by subcutaneous injection before the start of each surgery. Mice were single housed following surgery[2]. E2 concentrations were quantified using a commercially available ELISA kit. (Arbor Assays, Ann Arbor, Michigan, USA).

**Preparation of conditioned medium**

THP-1 cells and M2 macrophages are incubated at 37℃ for 2 days and medium is collected and centrifuged at 800rpm for 5 minutes. Supernatant is mixed with freshly prepared medium contained 10% FBS at 1 to 2 ratio for preparing conditioned medium. After 72 hours of incubation, medium collected from GFP-P53 overexpressed M2 macrophages or H1299 cancer cells are centrifuged at 800rpm for 5 minutes, and supernatant is mixed with freshly prepared medium contained 10% FBS at 1 to 2 ratio for preparing conditioned medium.

**Bisulfite sequencing assay**

Genomic DNA from A549 and E2A549 cells was purified by using QIAamp DNA mini kit (QIAGEN). Methylation status of purified DNA was analyzed by EZ DNA methylation kit (Zymo research) and bisulfite conversion was performed according to manufacturer’s instruction. -942 to -1099 nucleotide upstream transcription start site was reported the core region of p53 promoter [3]. Sequences containing these interested CpG sites were amplified by PCR, and primers were choosing by MethPrimer. Forward primer sequences were 5′-GTAGGTAGAAGATTTTCGGGA-3′, and reverse primer sequence were 5′-GCGAAATCTAATCCGAAATACG-3′. After amplification, PCR product was extracted by using Gel extraction miniprep system (Viogene), ligated into yT&A cloning vector (Yeastern biotech), and amplified by competent cells. Clones were containing sequences generated from estrogen treated A549 cells.

**References**

1 Wang YC, Wu YS, Hung CY, Wang SA, Young MJ, Hsu TI *et al*. USP24 induces IL-6 in tumor-associated microenvironment by stabilizing p300 and beta-TrCP and promotes cancer malignancy. *Nat Commun* 2018; 9: 3996.

2 Young M-J, Chen Y-C, Wang S-A, Chang H-P, Yang W-B, Lee C-C *et al*. Estradiol-mediated inhibition of Sp1 decreases miR-3194-5p expression to enhance CD44 expression during lung cancer progression. *Journal of Biomedical Science* 2022; 29: 3.

3 Tekpli X, Landvik NE, Anmarkud KH, Skaug V, Haugen A, Zienolddiny S. DNA methylation at promoter regions of interleukin 1B, interleukin 6, and interleukin 8 in non-small cell lung cancer. *Cancer immunology, immunotherapy : CII* 2013; 62: 337-345.

**Supplementary Figures**

**
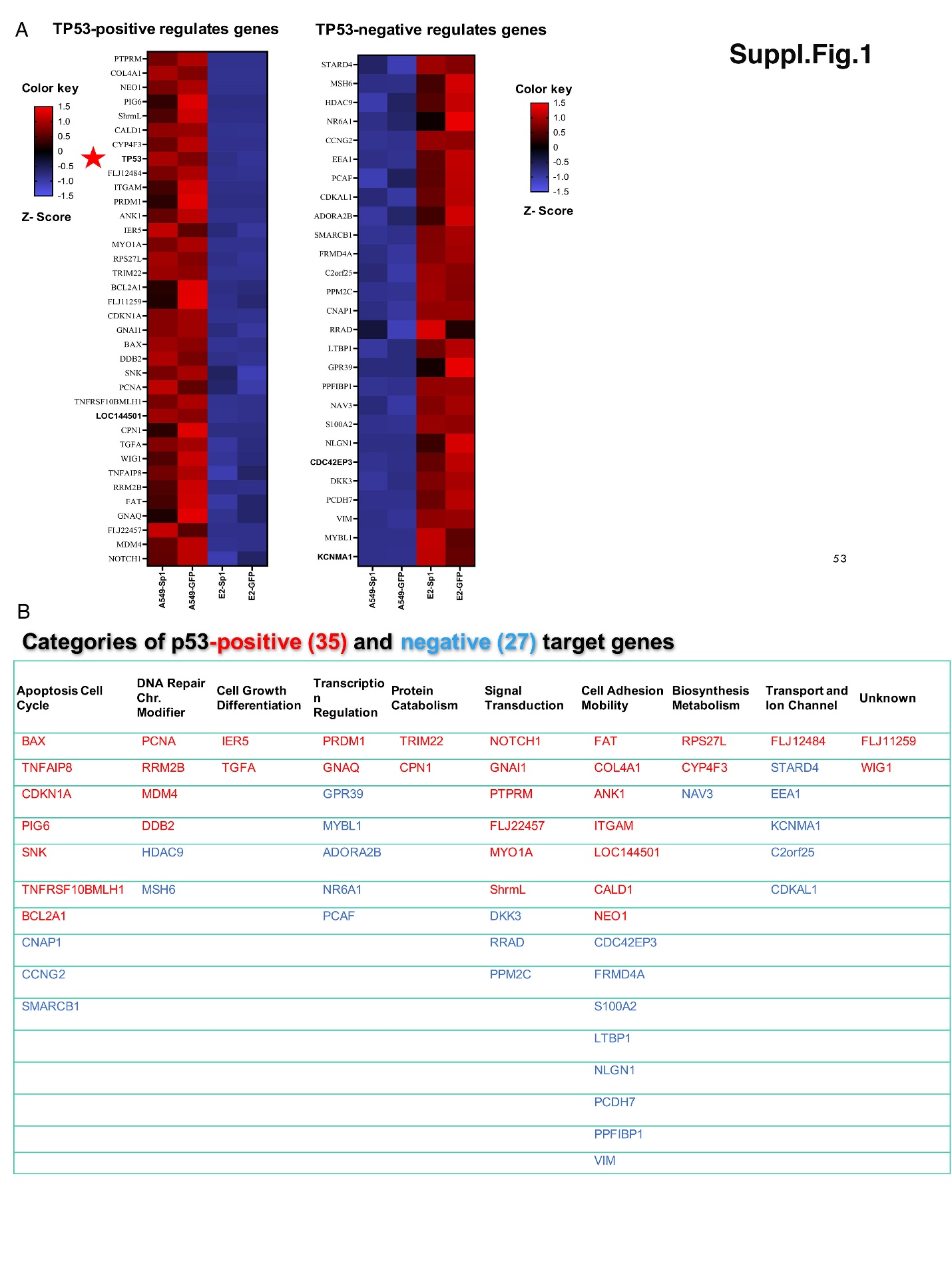
**

**Supplementary Fig. 1.** The gene expression profile of A549 lung cancer cells with or without E2 treatment or GFP-Sp1 expression. The mRNA levels of TP53 and its target genes regulated by E2 are shown here as a heatmap (A) and show the p53 target genes involved in various signaling pathways (B).


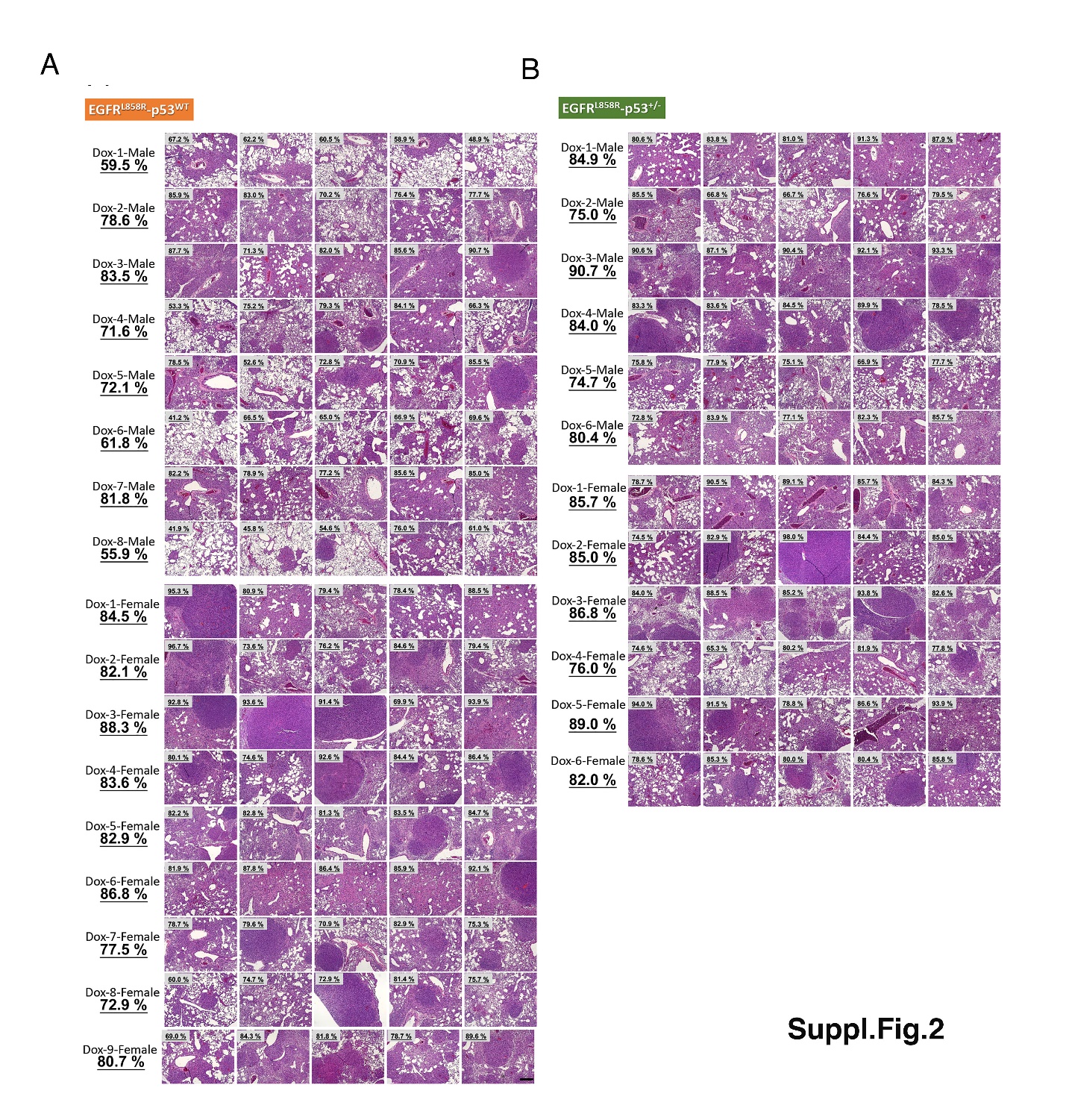


**Supplementary Fig. 2.** The effect of p53 on tumor formation in females was studied in female mice with (A) EGFR^L858R^- and (B) EGFR^L858R^ x TP53^-/+^-induced lung cancer following doxycycline treatment for 6 weeks. Mice were then sacrificed for H&E staining to determine the tumor area.


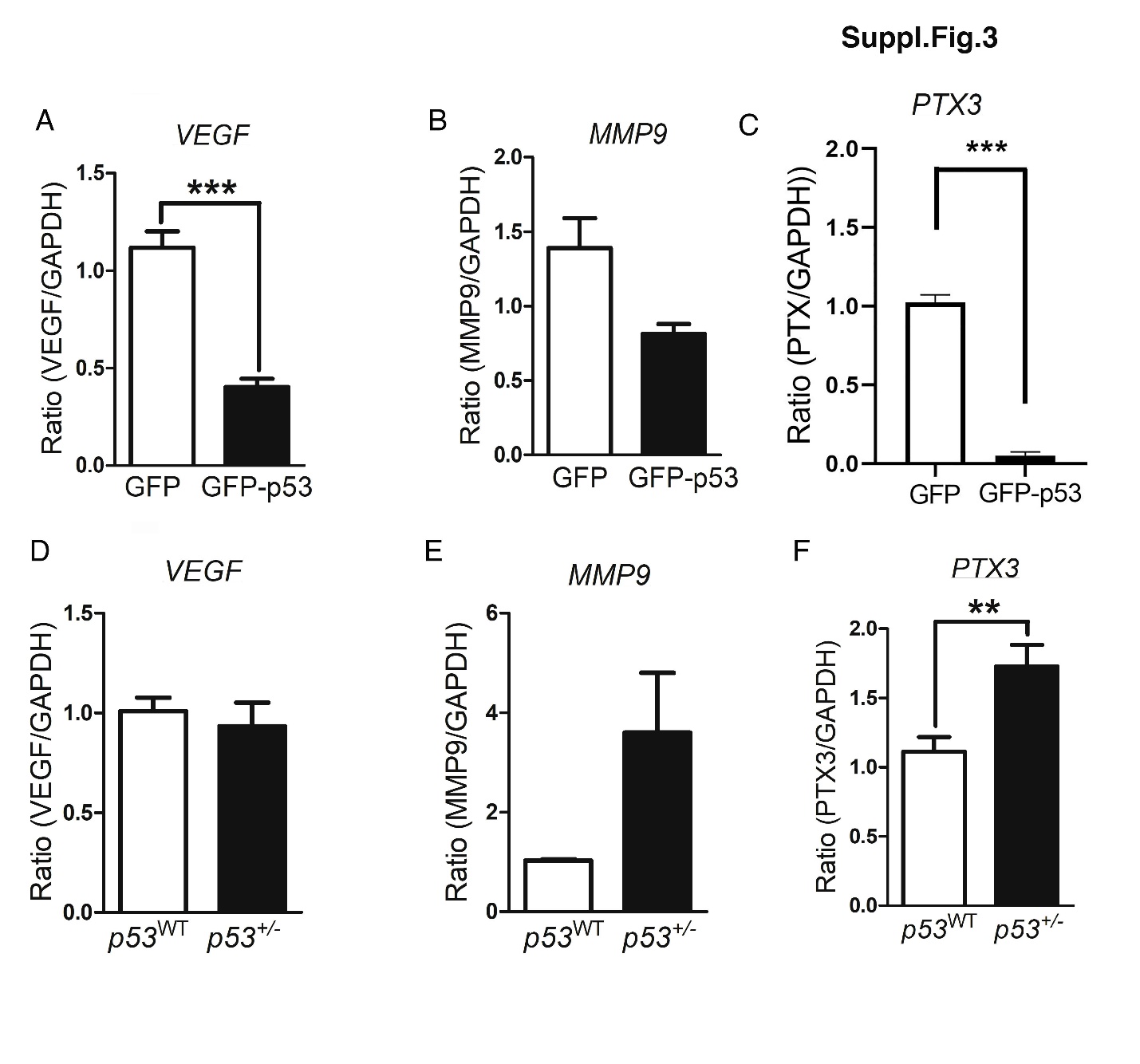


**Supplementary Fig. 3.** The mRNA levels of VEGF (A and D), MMP9 (B and E) and PTX3 (C and F) in GFP-p53-overexpressing H1299 cells and in mice with EGFR^L858R^-induced lung cancer mice with or without p53 knockout were studied by qPCR.

**Supplementary Tables**

| Name | Primer sequence |
| --- | --- |
| GDF15 (human) | Forward: 5’- CCAGGACGATAAGACCGTGT-3’ |
|  | Reverse: 5’- AGCCCATGTCCTTGAAGTTG -3’ |
| GDF15 (mouse) | Forward: 5’- CTTGAAGACTTGGGCTGGAG -3’ |
|  | Reverse: 5’- TAAGAACCACCGGGGTGTAG -3’ |
| FGF2 (human) | Forward: 5’- AGAGCGACCCTCACATCAAG -3’ |
|  | Reverse: 5’- ACTGCCCAGTTCGTTTCAGT -3’ |
| FGF2 (mouse) | Forward: 5’- AGCGGCTCTACTGCAAGAAC -3’ |
|  | Reverse: 5’- GCCGTCCATCTTCCTTCATA -3’ |
| CCL5 (human) | Forward: 5’- CGCTGTCATCCTCATTGCTA -3’ |
|  | Reverse: 5’- CCAGACTTGCTGTCCCTCTC -3’ |
| CCL5 (mouse) | Forward: 5’- CCCTCACCATCATCCTCACT -3’ |
|  | Reverse: 5’- CCTTCGAGTGACAAACACGA -3’ |
| VEGF-A (human) | Forward: 5’- CCCACTGAGGAGTCCAACAT -3’ |
|  | Reverse: 5’- TTTCTTGCGCTTTCGTTTTT -3’ |
| VEGF-A (mouse) | Forward: 5’- CAGGCTGCTGTAACGATGAA -3’ |
|  | Reverse: 5’- TTTCTTGCGCTTTCGTTTTT -3’ |
| MMP9 (human) | Forward: 5’- TTGACAGCGACAAGAAGTGG -3’ |
|  | Reverse: 5’- GCCATTCACGTCGTCCTTAT -3’ |
| MMP9 (mouse) | Forward: 5’- CGTCGTGATCCCCACTTACT -3’ |
|  | Reverse: 5’- AACACACAGGGTTTGCCTTC -3’ |
| PTX (human) | Forward: 5’- GTGGGTGGAGAGGAGAACAA -3’ |
|  | Reverse: 5’- TTCCTCCCTCAGGAACAATG -3’ |
| PTX (mouse) | Forward: 5’- GTGGGTGGAAAGGAGAACAA -3’ |
|  | Reverse: 5’- GGCCAATCTGTAGGAGTCCA -3’ |

**Supplementary Table 1.** All the primers used in this study were listed here.


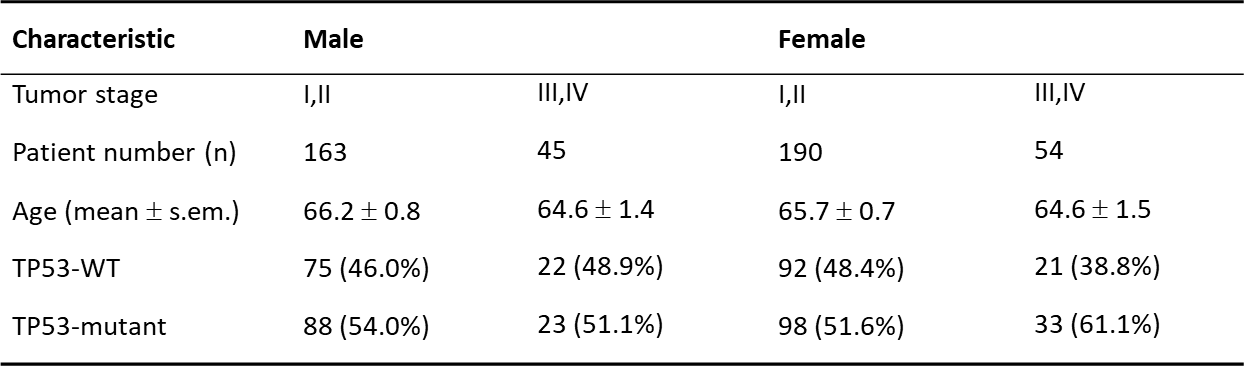


**Supplementary Table 2.** All the histories of patients used in Fig.2C were listed here.

**
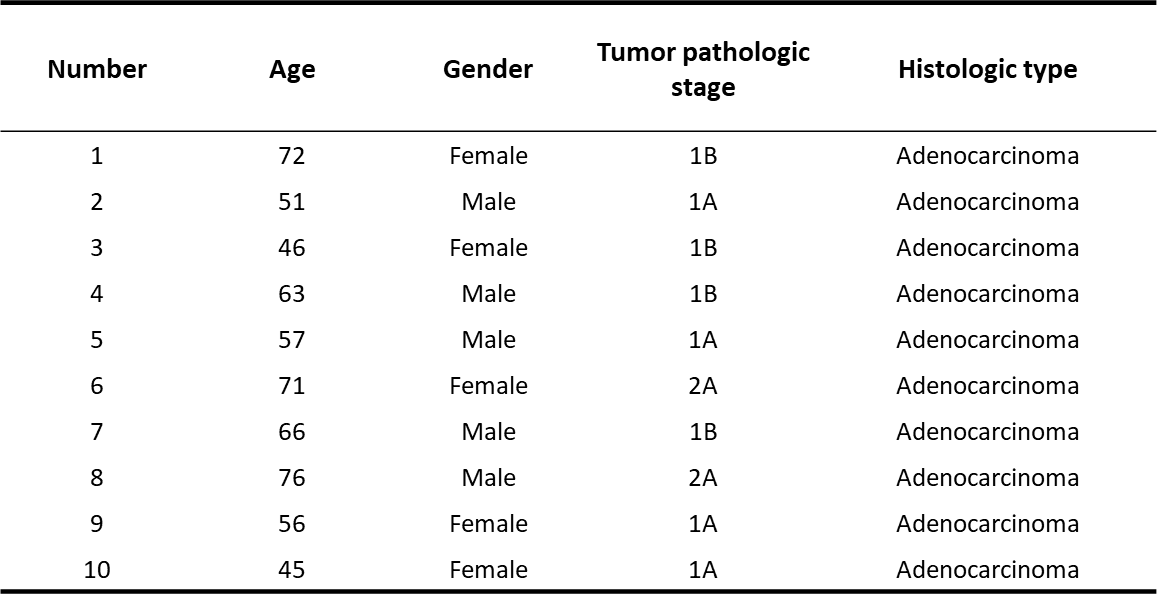
**

**Supplementary Table 3.** All the histories of patients used in Fig.8A were listed here.
